# Knowledge and Perceptions of COVID-19 Among Health Care Workers: Cross-Sectional Study

**DOI:** 10.2196/19160

**Published:** 2020-04-30

**Authors:** Akshaya Srikanth Bhagavathula, Wafa Ali Aldhaleei, Jamal Rahmani, Mohammadjavad Ashrafi Mahabadi, Deepak Kumar Bandari

**Affiliations:** 1 Institute of Public Health College of Medicine and Health Sciences United Arab Emirates University Abu Dhabi United Arab Emirates; 2 Department of Gastroenterology Sheikh Shakhbout Medical City Abu Dhabi United Arab Emirates; 3 Student Research Committee, National Nutrition and Food Technology Research Institute Department of Community Nutrition, Faculty of Nutrition and Food Technology Shahid Beheshti University of Medical Sciences Tehran Iran; 4 Iran University of Medical Sciences Tehran Iran; 5 Department of Clinical Pharmacy Vaagdevi Institute of Pharmaceutical Sciences Warangal, Telangana India

**Keywords:** coronavirus, outbreak, COVID-19, knowledge, perception, health care, questionnaire, health care worker

## Abstract

**Background:**

During the first week of March, the coronavirus disease 2019 (COVID-19) outbreak reached more than 100 countries with over 100,000 cases. Health care authorities have already initiated awareness and preparedness activities worldwide. A poor understanding of the disease among health care workers (HCWs) may result in delayed treatment and result in the rapid spread of the infection.

**Objective:**

This study aimed to investigate the knowledge and perceptions of HCWs about COVID-19.

**Methods:**

A cross-sectional, web-based study was conducted among HCWs about COVID-19 during the first week of March 2020. A 23-item survey instrument was developed and distributed randomly to HCWs using social media; it required 5 minutes to complete. A chi-square test was used to investigate the level of association among variables, with significance set to *P*<.05.

**Results:**

Of 529 participants, a total of 453 HCWs completed the survey (response rate: 85.6%); 51.6% (n=234) were male, 32.1% (n=147) were aged 25-34 years, and most were doctors (n=137, 30.2%) and medical students (n=134, 29.6%). Most participants (n=276, 61.0%) used social media to obtain information on COVID-19. A significant proportion of HCWs had poor knowledge of its transmission (n=276, 61.0%) and symptom onset (n=288, 63.6%) and showed positive perceptions of COVID-19. Factors such as age and profession were associated with inadequate knowledge and a poor perception of COVID-19.

**Conclusions:**

As the global threat of COVID-19 continues to emerge, it is critical to improve the knowledge and perceptions of HCWs. Educational interventions are urgently needed to reach HCWs worldwide, and further studies are warranted.

## Introduction

Coronavirus (CoV) infections are emerging respiratory viruses that are known to cause illness ranging from the common cold to severe acute respiratory syndrome (SARS) [1]. CoV is a zoonotic pathogen that can be transmitted via animal-to-human and human-to-human interactions [2]. Multiple epidemic outbreaks occurred in 2002 (SARS), with approximately 800 deaths, and in 2012 (Middle East respiratory syndrome coronavirus, MERS-CoV), with 860 deaths [2,3]. About 8 years after the MERS-CoV epidemic, the current outbreak of coronavirus disease 2019 (COVID-19) in Wuhan City, Hubei Province, China, has emerged as a global outbreak and significant public health issue [4]. On January 30, 2020, the World Health Organization (WHO) declared COVID-19 a public health emergency of international concern [5]. Astonishingly, during the first week of March, a devastating number of new cases were reported globally, and COVID-19 emerged as a pandemic. As of March 12, 2020, more than 125,000 confirmed cases across 118 countries and over 4600 deaths had been reported [6].

COVID-19 is spread by human-to-human transmission through droplet, feco-oral, and direct contact and has an incubation period of 2-14 days [7]. To date, no antiviral treatment or vaccine has been explicitly recommended for COVID-19. Therefore, applying preventive measures to control COVID-19 infection is the most critical intervention. Health care workers (HCWs) are the primary sector in contact with patients and are an important source of exposure to infected cases in health care settings; thus, HCWs are expected to be at high risk of infection. By the end of January, the WHO and Centers for Disease Control and Prevention (CDC) had published recommendations for the prevention and control of COVID-19 for HCWs [8,9]. The WHO also initiated several online training sessions and materials on COVID-19 in various languages to strengthen preventive strategies, including raising awareness and training HCWs in preparedness activities [10]. In several instances, misunderstandings among HCWs have delayed controlling efforts to provide necessary treatment [11], led to the rapid spread of infection in hospitals [12,13], and put patients' lives at risk.

Knowledge can influence the perceptions of HCWs due to their past experiences and beliefs [14-16]. Indeed, it can delay recognition and handling of potential COVID-19 patients during the pandemic period. However, the level of knowledge and perceptions of HCWs toward COVID-19 remain unclear. In this regard, the COVID-19 pandemic offers a unique opportunity to investigate the level of knowledge and perceptions of HCWs during this global health crisis. In addition, we aim to explore HCWs’ source of information of COVID-19 during this peak period.

## Methods

### Survey Instrument and Dissemination

A web-based, cross-sectional study was conducted using a survey instrument to obtain responses from HCWs globally during the first week of March 2020.

A 23-item survey instrument was developed using WHO course materials on emerging respiratory viruses, including COVID-19 [17]. The survey covered HCWs’ characteristics, awareness, information sources, and knowledge and perceptions related to COVID-19. The developed draft survey instrument was made accessible through a link and was distributed to 10 experts from different geographic regions to comprehensively assess the content domains of the questionnaire (using a scale of 1-5 points and encouraged open commentaries). In addition, the materials used for developing the survey questionnaire were also provided for any further clarifications. Moreover, to assess readability, 10 randomly selected faculty members read the questionnaire for 15 minutes and rated the ease of readability of the questionnaire ranging from 0 to 100 (0-30: confusing; 31-50: difficult; 51-70: standard; 70-90: easy; and 90-100 very easy). The pilot web survey was then conducted among 10 randomly selected HCWs to assess clarity, relevance, and acceptability. Feasibility and time required to answer the survey were evaluated on another 5 participants. These participants were not included in the research.

Refinements were made as required to facilitate better comprehension and to organize the questions before the final survey was distributed to the study population through a URL link. Briefly, we used Telegram, a cloud-based instant messaging app, used by more than 200 million people every month. The “Clinical Updates” group was established on December 28, 2017 to provide the latest medical research updates. The group includes more than 2500 active members of HCWs all over the world. In the group, the survey link was advertised to the target population and was opened in March 2020 for 10 days.

### Content of the Survey Instrument and Scoring System

The survey instrument comprised 23 closed-ended questions and took approximately 5 minutes to complete. The 23-item questionnaire was divided into three parts: participant characteristics (3 items), awareness of COVID-19 (2 items), source of information (4 statements/4-point Likert scale: 1 [least used] to 4 [most used]), knowledge about symptoms of COVID-19-affected patients (2 items), different modes of transmission (2 items), precautions and risk prevention (3 items) and perceptions of COVID-19 (7 items/true or false questions) (Multimedia Appendix 1). We used Qualitrics [18], an online survey tool to distribute the survey, and participants were given 30 mins to read, comprehend, and answer all the questions.

Knowledge was assessed by questions focusing on COVID-19 etiology, signs and symptoms, transmission, and risk prevention. Each response was scored as “1” (correct) and “0” (wrong), with scores ranging from 1 to 7. A cutoff level of ≤4 was considered to indicate poor knowledge about COVID-19 whereas >4 was considered adequate knowledge about COVID-19.

Perceptions toward COVID-19 were assessed using 7 items, and each question was labeled as good (scored as “1”) or poor perception (scored as “0”). Scores ranged from 0 to 7. The participants’ perceptions are classified as good (score >5) or poor (score ≤5).

### Data Analysis

The obtained data were coded, validated, and analyzed using SPSS version 24 (IBM). Descriptive analysis was applied to calculate frequencies and proportions. The chi-square test was used to investigate the level of association among variables. A *P* value of less than .05 was considered statistically significant.

### Ethical Considerations

Confidentiality of personal information was maintained throughout the study by making participants' information anonymous and asking participants to provide honest answers. Eligible HCWs’ participation in this survey was voluntary and was not compensated. Electronic informed consent was shown on the initial page of the survey. The study was performed following the Declaration of Helsinki as revised in 2013. The study was conducted following the Checklist for Reporting Results of Internet E-Surveys (CHERRIES) guidelines [19] (Multimedia Appendix 2).

## Results

### Overview

A total of 529 HCWs participated, 453 of whom completed the study questionnaire (85.6% response rate), including 234 (51.6%) men and 219 (48.3%) women. Most participants were below 44 years of age (n=378, 82.4%). The majority of participants were doctors (n=137, 30.2%) or medical students (n=134, 29.6%) and were from Asia (n=308, 68%). Table 1 shows the sociodemographic characteristics of the participants. Almost all participants agreed that they had heard about COVID-19 (n=443, 97.8%), but only 44.1% (n=200) of them had the opportunity to attend lectures or discussions related to COVID-19.

### Source of Information

When we asked about participants’ source for reliable information about COVID-19, the primary sources mentioned were official government websites and social media (Table 2). Approximately 30% (n=134) of the participants reported that they used news media (TV/video, magazines, newspapers, and radio) and social media (Facebook, Twitter, WhatsApp, YouTube, Instagram, Snapchat) to obtain information about COVID-19. Moreover, nearly 40% (n=179) of the participants sometimes discussed COVID-19-related topics with family and friends.

### Knowledge About COVID-19

Table 3 shows the level of knowledge about COVID-19 among HCWs. We identified significant knowledge gaps between doctors and other HCWs. For instance, 90 doctors (65.7%) and 176 allied health workers (55.7%) thought that COVID-19 originated from bats. The majority of the HCWs (n=338, 85.6%) agreed that maintaining hand hygiene, covering the nose and mouth while coughing, and avoiding sick patients could help to prevent COVID-19 transmission. Most doctors agreed that COVID-19 could lead to pneumonia, respiratory failure, and death (n=115, 84%; *P*=.04) and that supportive care is the only treatment option that is currently available (n=114, 83.2%; *P*<.001). However, participants' knowledge of questions related to the mode of transmission and the incubation period of COVID-19 was poor.

**Table 1:** Sociodemographic characteristics of health care workers (N=453).

Characteristic		Participants, n (%)
**Sex**		
	Male	234 (51.6)
	Female	219 (48.3)
**Age range (years)**		
	<25	145 (31.6)
	25-34	147 (32.1)
	35-44	86 (18.7)
	45-54	47 (10.2)
	55-64	28 (6.1)
**Occupation**		
	Doctor	137 (30.2)
	Medical student	134 (29.6)
	Pharmacist	61 (13.5)
	Academic doctor	61 (13.5)
	Nurse	24 (5.3)
	Lab technician	22 (4.9)
	Dentist	14 (3.1)
**Location**		
	Asia	308 (68)
	Africa	72 (15.9)
	Europe	40 (8.8)
	North America	11 (2.4)
	South America	7 (1.5)
	Unspecified	13 (2.9)
**Heard about COVID-19^a^**		
	Yes	443 (97.8)
	No	10 (2.2)
**Attended lectures or discussions about** **COVID-19**		
	Yes	200 (44.1)
	No	253 (55.8)

^a^COVID-19: coronavirus disease 2019.

**Table 2:** Participants’ sources for reliable information about coronavirus disease 2019 (COVID-19) (N=453).

Response	Source of COVID-19 information			
	News media, n (%)	Social media, n (%)	Government websites, n (%)	Family and friends, n (%)
Least used	134 (29.56)	139 (30.62)	151 (33.41)	53 (11.73)
Sometimes	139 (30.72)	139 (30.62)	101 (22.51)	91 (20.00)
More often	115 (25.40)	104 (22.97)	121 (26.71)	179 (39.51)
Most used	65 (14.34)	72 (15.9)	78 (17.21)	129 (28.47)

**Table 3:** Knowledge about coronavirus disease 2019 (COVID-19) among health care workers (N=453).

Question	Doctors (n=137), n (%)	Allied health workers (n=316), n (%)	Total correct responses, n (%)	*P* value^a^
COVID-19 is thought to originate from bats	90 (65.7)	176 (55.7)	266 (58.7)	.046
COVID-19 is transmitted through air, contact, fecal-oral routes	50 (36.5)	127 (40.2)	177 (39)	.46
Headache, fever, cough, sore throat, and flu are symptoms of COVID-19	109 (79.6)	223 (70.6)	332 (73.2)	.046
The incubation period of COVID-19 (2-14 days)	62 (45.3)	103 (32.6)	165 (36.4)	.01
COVID-19 leads to pneumonia, respiratory failure, and death	115 (84)	238 (75.3)	353 (77.9)	.04
Supportive care is the current treatment for COVID-19	114 (83.2)	193 (61)	307 (67.7)	.001
Hand hygiene, covering nose and mouth while coughing, and avoiding sick contacts can help in the prevention of COVID-19 transmission	117 (85.4)	271 (85.6)	388 (85.6)	.96

^a^*P*<.05 considered statistically significant between the groups.

### Perceptions About COVID-19

Over 78% (n=353) of the HCWs exhibited a positive perception of COVID-19. The majority of HCWs knew that sick patients should share their recent travel history (n=420, 92.7%), that flu vaccination is not sufficient to prevent COVID-19 (n=411, 90.7%), and that COVID-19 is not fatal (n=401, 88.5%). In addition, 87% (n=394) felt that washing hands with soap and water could help to prevent COVID-19 transmission; 84.3% (n=394) knew that symptoms appear in 2-14 days; and 85.6% (n=388) agreed that all equipment used in wet markets should be cleaned every day. However, 20.9% (n=95) of HCWs answered “no” when asked about eating well-cooked meat during the outbreak (Table 4).

Items related to perceptions of COVID-19 among HCWs were analyzed separately using a chi-square test to examine their association with age and sex and across different professions (Table 5).

**Table 4:** Health care workers’ perceptions toward coronavirus disease 2019 (COVID-19) (N=453).

Statement	Yes, n (%)	No, n (%)
COVID-19 symptoms appear in 2-14 days	394 (84.3)^a^	71 (15.6)
COVID-19 is fatal	52 (11.4)	401 (88.5)^a^
Flu vaccination is sufficient for preventing COVID-19	42 (9.2)	411 (90.7)^a^
During the outbreak, eating well-cooked and safely handled meat is safe	358 (78.1)^a^	95 (20.9)
Sick patients should share their recent travel history with health care providers	420 (92.7)^a^	33 (7.3)
Disinfect equipment and working area in wet markets at least once a day	388 (85.6)^a^	65 (14.3)
Washing hands with soap and water can help in the prevention of COVID-19 transmission	394 (87)^a^	59 (13)

^a^Indicates the correct answer.

**Table 5:** Association between respondent characteristics and perceptions of coronavirus disease 2019 (COVID-19).

Question and response			Sex						Age								Profession						
			Male (n=234), n (%)		Female (n=219), n (%)		*P* value^a^		<25 years (n=145), n (%)		25-44 years (n=233), n (%)		45-65 years (n=75), n (%)		*P* value^a^		Doctors (n=137), n (%)		Medical students (n=134), n (%)		Others (n=182), n (%)		*P* value^a^
**COVID-19 symptoms appear in 2-14 days**							.75								*.001*								*.011*
	Yes^b^	198 (84.6)		183 (83.5)				130 (89.6)		207 (88.8)		39 (52)				126 (92)		116 (86.5)		146 (80.2)			
	No	36 (15.3)		36 (16.4)				15 (10.4)		26 (11.1)		36 (48)				11 (8)		18 (13.5)		36 (19.8)			
**COVID-19 is fatal**							.19								.78								.31
	Yes	22 (9.4)		29 (13.2)				127 (87.5)		207 (88.8)		68 (90.6)				116 (84.6)		112 (83.5)		143 (78.5)			
	No^b^	212 (90.6)		190 (86.7)				18 (12.5)		26 (11.1)		7 (9.4)				21 (15.4)		22 (16.5)		39 (21.5)			
**Flu vaccinated is sufficient for preventing COVID-19**							.94								.07								.11
	Yes	24 (10.2)		22 (10.1)				21 (14.5)		19 (8.1)		5 (6.6)				18 (13.1)		16 (12)		36 (19.8)			
	No^b^	210 (89.7)		197 (89.9)				124 (85.5)		214 (91.9)		70 (93.4)				119 (86.9)		118 (88)		146 (80.2)			
**During the outbreak, eating well-cooked and safely handled meat is safe**							.67								.13								*.099*
	Yes^b^	192 (82)		183 (83.5)				113 (77.9)		200 (85.8)		63 (84)				114 (83.2)		98 (73.1)		136 (74.7)			
	No	42 (18)		36 (16.4)				32 (22)		33 (14.2)		12 (16)				23 (16.8)		36 (26.9)		46 (25.3)			
**Sick patients should share their recent travel history with health care providers**							.84								.51								*.02*
	Yes^b^	228 (97.4)		214 (97.7)				141 (97.2)		229 (98.2)		72 (96)				131 (95.6)		124 (92.5)		158 (86.8)			
	No	6 (2.6)		5 (2.3)				4 (2.8)		4 (1.8)		3 (4)				6 (4.4)		10 (7.5)		24 (13.2)			
**Disinfect equipment’s and working area in wet markets at least once a day**							.26								.54								.41
	Yes^b^	205 (87.6)		199 (90.8)				131 (90.3)		206 (88.4)		64 (85.3)				116 (84.6)		117 (87.3)		149 (81.8)			
	No	29 (12.4)		20 (9.2)				14 (9.7)		27 (11.6)		11 (14.7)				21 (15.4)		17 (12.7)		33 (18.2)			
**Washing hands with soap and water can help in prevention of COVID-19 transmission**							.58								.24								.88
	Yes^b^	204 (87.2)		187 (85.3)				120 (82.7)		207 (88.8)		65 (86.6)				118 (86.1)		116 (86.5)		160 (87.9)			
	No	30 (12.8)		32 (13.6%)				25 (17.3)		26 (11.1)		10 (13.4)				19 (13.9)		18 (13.5)		22 (12.1)			

^a^Significant at *P*<.05 (italicized) between the groups.

^b^Indicates the correct answer.

Nearly 90% (n=130) of the youngest participants (<25 years) and 92% (n=126) of the doctors believed that the symptoms of COVID-19 appeared as early as 2-14 days; the differences among the respondent groups were statistically significant (*P*<.001). Moreover, a significant proportion of doctors perceived eating well-cooked/handled meat to be safe (n=114, 83.2%). Medical students were found to have the perception that flu vaccination is not sufficient to prevent COVID-19 transmission (n=118, 88%). A large number of allied health workers incorrectly believed that it is not safe to eat well-processed meat during the COVID-19 outbreak (n=46, 25.3%), that COVID-19 is fatal (n=143, 78.5%), that there is a delay in symptoms (n=36, 19.8%), and that flu vaccination is sufficient (n=36, 19.8%) compared with other participants in the respective groups.

## Discussion

### Principal Findings

At present, COVID-19 is a global topic of discussion in the media and among the public, especially among HCWs and patients. With the currently mounting COVID-19 transmission raising tensions for everyone, including for health officials and health systems, an important question arises regarding how we manage information to help frontline HCWs in times of public health crisis. For this reason, we investigated HCWs’ knowledge and perceptions of the prevention and control of COVID-19 at the pandemic level.

Knowledge and perceptions of COVID-19 varied across different categories of HCWs. Our study revealed that HCWs have insufficient knowledge about COVID-19 but showed positive perceptions of COVID-19 transmission prevention. We also found that more than 33% (n=151) of HCWs used official government websites as a primary source of information about COVID-19. This indicates that the COVID-19-related updates posted online by official government health authorities had positive implications for improving HCWs’ knowledge levels. Obtaining information from authentic sources is pivotal for disseminating unbiased and reliable data about the emerging COVID-19 infection and is essential for HCWs’ preparedness and response. However, a finding of considerable concern is that more than 61% (n=278) of HCWs used social media as a source of information. Currently, there is a vast diversity of information available through the internet, including unverified malicious information, that can spread quickly and misguide HCWs. In particular, health authorities and scientists have warned that widespread misinformation about COVID-19 is a serious concern causing xenophobia worldwide [4,20-22]. In this regard, HCWs should carefully evaluate COVID-19-related information and should use scientific and authentic content as information sources.

The findings of this study suggest a significant gap between the amount of information available on COVID-19 and the depth of knowledge among HCWs, particularly about the mode of transmission and the incubation period of COVID-19. Additionally, many allied health workers had inaccurate knowledge of COVID-19 (eg, can be treated with antivirals and that there is a vaccine available). This is unfortunate because the surge of COVID-19 is globally devastating, and a large number of resources are provided by health care authorities to educate HCWs and improve their knowledge of COVID-19. One possible explanation for these differences in knowledge is that doctors are more educated in infectious diseases and pharmacotherapy because of their continuous professional development. Therefore, our findings suggest that greater encouragement from health authorities is needed to distribute COVID-19-related knowledge to all categories of HCWs.

Generally, most participants had a positive perception of the prevention and control of COVID-19. However, discrepancies were identified in the perceptions of different categories of HCWs. For instance, only half (n=32, 52%) of the HCWs aged 45-65 years believed that the symptoms of COVID-19 appeared as early as 2 or as late as 14 days (*P*<.001). If these responses are truly representative of HCWs, this could have adverse consequences on patient care and also on the dynamics of potential COVID-19 outbreaks. This apparent lack of knowledge could result in delays in the implementation of necessary confinement measures and personal protective equipment, which may increase the burden of COVID-19. In our study, more than a quarter of the medical students thought that eating meat during the outbreak was unsafe. This may be due to the fact that COVID-19 was closely linked to a wet market in China and other viral diseases such as SARS, MERS, and Ebola emerged from zoonotic origins [23-25]. Thus, people often believe that the consumption of undercooked meat may enhance viral transmission. However, further investigation is still required. Approximately 20% (n=36) of allied health workers believed that the flu vaccine is sufficient for COVID-19 prevention. Finally, a vast majority of HCWs strongly agreed that maintaining hygiene activities, reporting recent travel history when individuals are sick, and cleaning the equipment used in wet markets are strongly recommended.

### Limitations

We used WHO training material for the detection, prevention, response, and control of COVID-19 to develop a validated questionnaire. The developed questionnaire was pilot tested, and open-ended questions were limited to reduce information bias.

However, this study has some limitations that should be considered. This is a cross-sectional study conducted online among HCWs during a time (ie, first week of March 2020) when an alarming number of cases were being reported globally; this might limit generalizations. In addition, the data presented in this study are self-reported and partly dependent on the participants' honesty and recall ability; thus, they may be subject to recall bias. Lastly, due to the 4-week closure of higher educational institutions in the United Arab Emirates during the COVID-19 outbreak [26], the institutional review board was not approached. Despite these limitations, our findings provide valuable information about the knowledge and perceptions of HCWs during a peak period of the pandemic.

### Conclusion

We identified a significant gap in information source, poor knowledge levels, and discrepancies in perceptions of COVID-19 among our study participants. As the global threat of COVID-19 continues to emerge, greater efforts through educational campaigns that target HCWs and the wider population beyond borders are urgently needed.

## Appendix

Multimedia Appendix 1 Survey questionnaire.

Multimedia Appendix 2 Checklist for Reporting Results of Internet E-Surveys (CHERRIES).

## Abbreviations

CDC: Centers for Disease Control and Prevention
CHERRIES: Checklist for Reporting Results of Internet E-Surveys
CoV: coronavirus
COVID-19: coronavirus disease 2019
HCW: health care worker
MERS-CoV: Middle East respiratory syndrome coronavirus
SARS: severe acute respiratory syndrome
WHO: World Health Organization
