# Supplementary material for: Knowledge and Perceptions of COVID-19 Among Health Care Workers: Cross-Sectional Study
Source: JMIR Public Health Surveill. 2020 Apr 30;6(2):e19160. doi: 10.2196/19160 (PMC7193987; doi:10.2196/19160)
Supplement: Multimedia Appendix 1 [file publichealth_v6i2e19160_app1.docx]

**Survey on Novel Coronavirus: What we know about it?**

Dear health professional,

The current Coronavirus disease (COVID-19) epidemic is devastating. It has been declared an "Emergency of International Concern" by the World Health Organization (WHO). The number of COVID-19 cases was consistently increasing around the world. As the health professional's knowledge is of utmost importance to prevent and control the spreading of COVID-19. In this regard, we would likely to know the level of knowledge and Perceptions of health professionals about COVID-19, this can provide a greater opportunity to understand the existing gaps about the COVID-19 and to scale up the interventional strategies. Thus, we invite you to participate in this short survey and provide your valuable opinion regarding COVID-19.

This survey constitutes 15 questions that are focused on investigating the various domains of knowledge and perception about COVID-19. We request you to provide honest responses to the questions provided.

By clicking the below link, you agree to participate voluntarily in this survey and given your consent to use your anonymous data for research.

You may exit the survey at any stage, and no compensation will be provided.

If you have any further queries, please write to me @ [akshaypharmd@gmail.com](mailto:akshaypharmd@gmail.com)

Thanking you in advance

Sincerely

Dr. Akshaya Srikanth Bhagavathula

Novel Coronavirus (SAR-COV-19): What we know about it?

Sociodemographic characteristics

Q1 What is your gender

- Male (1)
- Female (2)

Q2 What is your age (years)

- 18 - 24 (2)
- 25 - 34 (3)
- 35 - 44 (4)
- 45 - 54 (5)
- 55 - 64 (6)
- 65 - 74 (7)
- >74 (60)

Q3 What is your profession

________________________________________________________________

Q4 Have you heard of Novel Coronavirus

- Yes (1)
- No (2)

Q6 Had you attended any of the lectures/discussions about Novel Coronavirus disease ?

- Yes (1)
- No (2)

Q7 On a scale of 1 "least used sources" to 4 "most used sources", how do you rank your sources of information about the Wuhan Novel Coronavirus (SAR-COV-19) outbreak in China as global emergency?

|  | Least used (1) | Sometimes (2) | More often (3) | Most used (4) |
| --- | --- | --- | --- | --- |
| News, Media (TV, Radio, newspapers etc.., (1) |  |  |  |  |
| Social media (Facebook, Twitter, Whatsapp, YouTube, Instagram, Snapchat…) (2) |  |  |  |  |
| Official government websites (MOH, DHA, DOH, WHO, CDC…..) (3) |  |  |  |  |
| Family member, colleague or friend (4) |  |  |  |  |

| Page Break |  |
| --- | --- |

Please answer the following questions

Q12 What is the incubation period of Novel coronavirus (SAR-COV-19)?

- 2-7 days
- **2-14 days**
- 7-14 days
- 7-21 days
- None of the above

| 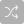 |
| --- |

Q13 Symptoms of novel coronavirus (SAR-COV-19) are all except:

- Headache (1)
- Fever (2)
- Cough (3)
- Sore throat and runny nose (4)
- **Skin rash (5)**

| 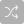 |
| --- |

Q14 Novel Coronavirus (SAR-COV-19) origin is thought to be from:

- **Bats (1)**
- Snakes (2)
- Fish (3)
- Camel (4)
- Unknown (5)

Q15 Novel Coronavirus (SAR-COV-19) transmission occur through:

- Air (1)
- Contact (2)
- Feco-oral (3)
- **All the above (4)**
- None of the above (5)

Q16 What are the complications of Novel Coronavirus (SAR-COV-19)?

- Pneumonia (1)
- Respiratory failure (2)
- Death (3)
- **All the above (4)**

Q17 What is the treatment of Novel coronavirus (SAR-COV-19)?

- **Supportive care (1)**
- Antiviral therapy (2)
- Vaccination (3)
- None of the above (4)

Q18 How to reduce the risk of transmission?

- Hand hygiene (1)
- Covering the nose and mouth when coughing (2)
- Avoiding sick contacts (3)
- Having well-cooked meat and eggs (4)
- **All of the above (5)**

| Page Break |  |
| --- | --- |

| 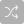 |
| --- |

Q21 Answer the following True or false questions

|  | True | False |
| --- | --- | --- |
| It is believed that symptoms of the novel coronavirus (SAR-COV-19) may appear in as few as 2 days or as long as 14 after exposure. (1) | - x |  |
| If anyone get the novel coronavirus (SAR-COV-19), there is no possibility of survival. (2) |  | - x |
| If anyone had a flu shot, a vaccinated against the novel coronavirus (SAR-COV-19) is sufficient (3) |  | - x |
| Even in areas experiencing outbreaks, meat products can be safely consumed if these items are cooked thoroughly and properly handled during food preparation. (4) | - x |  |
| If anyone has a fever, cough and difficulty breathing seek medical care early and share previous travel history with the health care providers. (5) | - x |  |
| If anyone work in a "wet market" it is recommended to disinfect the equipment and working area at least once a day (6) | - x |  |
| As per WHO guidelines for the novel coronavirus, you only need to wash your hands when they are visibly dirty. (7) | - x |  |

**********************************END****************************************
