# Supplementary material for: Knowledge and Perceptions of COVID-19 Among Health Care Workers: Cross-Sectional Study
Source: JMIR Public Health Surveill. 2020 Apr 30;6(2):e19160. doi: 10.2196/19160 (PMC7193987; doi:10.2196/19160)
Supplement: Multimedia Appendix 2 [file publichealth_v6i2e19160_app2.pdf]

	Checklist for Reporting Results of Internet E-Surveys (CHERRIES)		
Item Category	***Checklist Item***	***Explanation***	
**Design**			
	Describe survey design	Describe target population, sample frame. Is the sample a convenience sample? (In "open" surveys this is most likely.)	We wanted a broadly representative sample of the Healthcare workers globally. We used open survey via online platform.
**IRB (Institutional Review Board) approval and informed consent process**			
	IRB approval	Mention whether the study has been approved by an IRB.	Not obtained due to closure of institutions
	Informed consent	Describe the informed consent process. Where were the participants told the length of time of the survey, which data were stored and where and for how long, who the investigator was, and the purpose of the study?	Informed consent was presented in the first page of the survey that they can read and click approve to participate.
	Data protection	If any personal information was collected or stored, describe what mechanisms were used to protect unauthorized access.	No identifiable information was collected.
**Development and pre-testing**			
	Development and testing	State how the survey was developed, including whether the usability and technical functionality of the electronic questionnaire had been tested before fielding the questionnaire.	The survey went through 2 stages of development. The first phase sought relevant questionnaires and created new questions and was piloted with the team. Revised versions were tested in 15 participants. A revised version from this was then used in the main survey.
**Recruitment process and description of the sample having access to the questionnaire**			
	Open survey versus closed survey	An "open survey" is a survey open for each visitor of a site, while a closed survey is only open to a sample which the investigator knows (password-protected survey).	The survey was open to all the participants.
	Contact mode	Indicate whether or not the initial contact with the potential participants was made on the Internet. (Investigators may also send out questionnaires by mail and allow for Web-based data entry.)	The survey instrument was focused only on healthcare workers and was focused mainly on potential participants.
	Advertising the survey	How/where was the survey announced or advertised? Some examples are offline media (newspapers), or online (mailing lists – If yes, which ones?) or banner ads (Where were these banner ads posted and what did they look like?). It is important to know the wording of the announcement as it will heavily influence who chooses to participate. Ideally the survey announcement should be published as an appendix.	The survey was advertised using Telegram group "Clinical Updates" group containing 2500 members and was advertised from March 1-10, 2020.
**Survey administration**			
	Web/E-mail	State the type of e-survey (eg, one posted on a Web site, or one sent out	Web survey

		through e-mail). If it is an e-mail survey, were the responses entered manually into a database, or was there an automatic method for capturing responses?	
	Context	Describe the Web site (for mailing list/newsgroup) in which the survey was posted. What is the Web site about, who is visiting it, what are visitors normally looking for? Discuss to what degree the content of the Web site could pre-select the sample or influence the results. For example, a survey about vaccination on a anti-immunization Web site will have different results from a Web survey conducted on a government Web site	Qualtrics XM https://qtrial2019q2az1.az1.qualtrics.com/jfe/form/SV_06S35eN8J9Hm4bb
	Mandatory/voluntary	Was it a mandatory survey to be filled in by every visitor who wanted to enter the Web site, or was it a voluntary survey?	The survey participation is completely voluntary The people can have right to leave the survey at any time.
	Incentives	Were any incentives offered (eg, monetary, prizes, or non-monetary incentives such as an offer to provide the survey results)?	No compensation was provided
	Time/Date	In what timeframe were the data collected?	The data was collected after one week of initiating The survey from March 1 to March 7.
	Randomization of items or questionnaires	To prevent biases items can be randomized or alternated.	The questions were randomized.
	Adaptive questioning	Use adaptive questioning (certain items, or only conditionally displayed based on responses to other items) to reduce number and complexity of the questions.	Adaptive questioning was not applied.
	Number of Items	What was the number of questionnaire items per page? The number of items is an important factor for the completion rate.	All questions fit an average screen without scrolling. This averaged between 5 to 6 questions per page.
	Number of screens (pages)	Over how many pages was the questionnaire distributed? The number of items is an important factor for the completion rate.	The total survey was 3 pages.
	Completeness check	It is technically possible to do consistency or completeness checks before the questionnaire is submitted. Was this done, and if "yes", how (usually JAVAScript)? An alternative is to check for completeness after the questionnaire has been submitted (and highlight mandatory items). If this has been done, it should be reported. All items should provide a non-response option such as "not applicable" or "rather not say", and selection of one response option should be enforced.	All questions had to be completed. We did not use any consistency checks.
	Review step	State whether respondents were able to review and change their answers (eg, through a Back button or a Review step which displays a summary of the	Respondents were able to go back and review questions.

		responses and asks the respondents if they are correct).	
**Response rates**			85.6%
	Unique site visitor	If you provide view rates or participation rates, you need to define how you determined a unique visitor. There are different techniques available, based on IP addresses or cookies or both.	We used IP address to define a unique visitor.
	View rate (Ratio of unique survey visitors/unique site visitors)	Requires counting unique visitors to the first page of the survey, divided by the number of unique site visitors (not page views!). It is not unusual to have view rates of less than 0.1 % if the survey is voluntary.	N/A
	Participation rate (Ratio of unique visitors who agreed to participate/unique first survey page visitors)	Count the unique number of people who filled in the first survey page (or agreed to participate, for example by checking a checkbox), divided by visitors who visit the first page of the survey (or the informed consents page, if present). This can also be called "recruitment" rate.	The survey link was sent out in multiple waves to the participants until completion.<br>However, not all participants will have open the link, and some may have tried to start the survey but may have reached the quota.
	Completion rate (Ratio of users who finished the survey/users who agreed to participate)	The number of people submitting the last questionnaire page, divided by the number of people who agreed to participate (or submitted the first survey page). This is only relevant if there is a separate "informed consent" page or if the survey goes over several pages. This is a measure for attrition. Note that "completion" can involve leaving questionnaire items blank. This is not a measure for how completely questionnaires were filled in. (If you need a measure for this, use the word "completeness rate".)	Of the 529 individuals that clicked the link (85.6%) completed the survey.
**Preventing multiple entries from the same individual**			
	Cookies used	Indicate whether cookies were used to assign a unique user identifier to each client computer. If so, mention the page on which the cookie was set and read, and how long the cookie was valid. Were duplicate entries avoided by preventing users access to the survey twice; or were duplicate database entries having the same user ID eliminated before analysis? In the latter case, which entries were kept for analysis (eg, the first entry or the most recent)?	N/A
	IP check	Indicate whether the IP address of the client computer was used to identify potential duplicate entries from the same user. If so, mention the period of time for which no two entries from the same IP address were allowed (eg, 24 hours). Were duplicate entries avoided by preventing users with the same IP address access to the survey twice; or were duplicate database entries having the same IP address within a given period of time eliminated before	The team carefully checked for duplicate respondents. They check for multiple IP addresses.

		analysis? If the latter, which entries were kept for analysis (eg, the first entry or the most recent)?	
	Log file analysis	Indicate whether other techniques to analyze the log file for identification of multiple entries were used. If so, please describe.	N/A
	Registration	In “closed” (non-open) surveys, users need to login first and it is easier to prevent duplicate entries from the same user. Describe how this was done. For example, was the survey never displayed a second time once the user had filled it in, or was the username stored together with the survey results and later eliminated? If the latter, which entries were kept for analysis (eg, the first entry or the most recent)?	N/A
**Analysis**			
	Handling of incomplete questionnaires	Were only completed questionnaires analyzed? Were questionnaires which terminated early (where, for example, users did not go through all questionnaire pages) also analyzed?	Very few non-completers provided any information (stopping at the first page) so we did not compare completers to non-completer.
	Questionnaires submitted with an atypical timestamp	Some investigators may measure the time people needed to fill in a questionnaire and exclude questionnaires that were submitted too soon. Specify the timeframe that was used as a cut-off point, and describe how this point was determined.	30 minutes was allotted to finish the questionnaire
	Statistical correction	Indicate whether any methods such as weighting of items or propensity scores have been used to adjust for the non-representative sample; if so, please describe the methods.	We had no reason to expect differences in respondents and no data to allow us to adjust anyway.
